# The Individual and Interactive Effects of Climate, Land Use and Nutrient Enrichment on Macroinvertebrate Dynamics in South African Rivers

**DOI:** 10.1002/ece3.74252

**Published:** 2026-08-31

**Authors:** Buntu Fanteso, Tatenda Dalu, Ryan Wasserman, Wilbert Kadye, Pule Mpopetsi, Michelle Jackson

**Affiliations:** ^1^ Department of Biology University of Oxford Oxford UK; ^2^ Centre for Invasion Biology, School for Climate Studies Stellenbosch University Stellenbosch South Africa; ^3^ Department of Zoology and Entomology Rhodes University Makhanda South Africa; ^4^ School of Biology and Environmental Sciences University of Mpumalanga Nelspruit South Africa; ^5^ Department of Ichthyology and Fisheries Science Rhodes University Makhanda South Africa; ^6^ Somerville College University of Oxford Oxford UK

**Keywords:** climate change, freshwater ecosystems, land use, multiple stressors, nutrient enrichment

## Abstract

Freshwater ecosystems are increasingly degraded by multiple stressors arising from climate change and local anthropogenic pressures. However, the potentially important interactive effects of stressors are not well understood. While there is growing evidence of multiple stressor effects from experimental studies, there is a need for more field‐based studies to provide complementary insights where there is high biological variability and context dependencies. We conducted an extensive survey of benthic macroinvertebrate communities across 30 streams in South Africa to examine responses to both the single and interactive effects of climate, human land cover and nutrient enrichment. We sampled macroinvertebrates and physicochemical variables at each site during the cold–dry and hot–wet seasons. Land use was classified using the 2022 South African National Landcover (SANCL) data. We used temperature and rainfall data from the South African Weather Service (SAWS) as climate proxies. Ammonium was the most pervasive stressor, acting both independently and in interaction with climate and human land cover to influence macroinvertebrate community dynamics, including independent effects on Ephemeroptera, Plecoptera and Trichoptera (EPT) richness and mean biomass (mg) and in interaction with human land cover to negatively affect overall and EPT abundance. The interaction of human land cover and rainfall, as well as ammonium, rainfall and season interaction showed positive effects on mean body size, indicating that rainfall is an important stressor in modulating the effects. Overall, our study suggests that both the individual and interactive effects of climate and anthropogenic stressors shape freshwater macroinvertebrate community dynamics. Our study highlights that reducing nutrient inputs and effective land use management would go a long way towards conserving freshwater invertebrates.

## Graphical abstract

This study uses macroinvertebrate data from 30 streams in South Africa to understand how multiple climate‐ and anthropogenic‐related stressors shape their distribution. Both single and interactive terms shaped community composition, with stressor interactions dominating responses. Overall, we found context‐dependent interactions and highlight that managing land use and pollution remains a key focus of freshwater conservation.

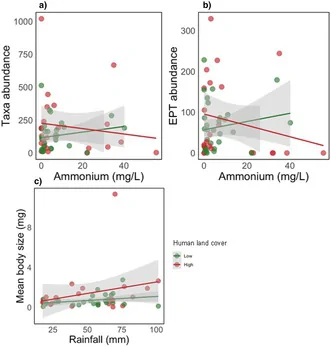

## 1 Introduction

Freshwater environments are increasingly exposed to multiple stressors associated with climate change, nutrient enrichment, and land‐use alterations (Fouchy et al. 2019; Lin et al. 2021; Oberdorff 2022), making them among the most threatened ecosystems on the planet (Saqira et al. 2024). These stressors modify habitat structure, resource availability and water quality, leading to shifts in the biological composition of freshwater ecosystems (Spears et al. 2022; Feio et al. 2023). Importantly, stressors rarely occur in isolation in freshwater ecosystems; instead, they commonly interact in complex ways, making it challenging to accurately predict ecological responses (Jackson et al. 2016; Macaulay et al. 2021; Brasseur et al. 2022). Understanding stressor interactions is, therefore, a central challenge in freshwater ecosystems and a growing priority for effective management and conservation decisions (Birk et al. 2020).

Land use change from human activities is the primary driver of global biodiversity loss (Cabernard et al. 2024; Ma et al. 2025). In freshwater ecosystems, human land use degrades habitats, resulting in shifts in community composition with effects that can vary seasonally (Matomela et al. 2021; Dalu et al. 2022; Gücker et al. 2024; Wang et al. 2025). Similarly, the elevated nutrient inputs associated with anthropogenic land use determine ecological conditions by promoting primary production, often leading to algal growth, hypoxia, and altering resource availability (Partani et al. 2024). Nutrient enrichment accounts for 35% of the variation in ecological status in European waters (Polazzo et al. 2022) and 34% in African rivers (Nkwasa et al. 2024). Climate change shifts thermal and hydrological patterns, affecting biomass and community dynamics (Woodward et al. 2010). These stressors can interact, leading to additive (combined effects of the stressors equal the sum of their individual effects), synergistic (combined effects are greater than additive) or antagonistic (combined effects are smaller than the additive effects) effects (Bao et al. 2024; Parker et al. 2024).

Despite progress in understanding multiple stressors in freshwater ecosystems, a recent systematic review of multiple‐stressor research highlights pronounced geographical biases, particularly in African freshwater ecosystems, which remain underrepresented in multiple‐stressor studies (Orr et al. 2024). There is also a lack of high‐order or three‐way interaction studies, and understanding the mechanisms for the complex interactions remains a challenge in ecological research (Taniwaki et al. 2017). The role of temporal dynamics, such as seasonal differences, in freshwater responses to multiple stressors also remains underexplored (Jackson et al. 2021; Segurado et al. 2021; He et al. 2023). Additionally, most research has relied on controlled experimental approaches, with limited evidence from field surveys, limiting our mechanistic understanding of stressor interactions across diverse and heterogeneous ecosystems (Emmons et al. 2025). Additional research from real‐world field surveys is therefore required to advance our understanding of multiple stressors in freshwater ecosystems, considering the significant variability and context dependencies in these environments.

A past study in South Africa found that river food webs varied across climatic and land‐use gradients (Jackson et al. 2020). However, the study did not account for nutrient enrichment, despite its strong influence on South African freshwater ecosystems (Adams et al. 2020; Mararakanye et al. 2022). We build on this work by quantifying nutrient enrichment (ammonium, phosphate, nitrate) as a stressor and using different response variables—we focus on macroinvertebrate community metrics (i.e., taxa abundance, taxa richness, Ephemeroptera, Plecoptera and Trichoptera (EPT) abundance, EPT richness, Simpson's diversity index, mean body size and biomass). We sample many of the same sites as Jackson et al. (2020) but also expand the spatial scale and scope by introducing new streams to gain a more comprehensive insight. We address the following broad questions: (i) do climate, human land cover and nutrient enrichment independently affect macroinvertebrate communities across a stressor gradient? and (ii) do climate, human land cover, and nutrient enrichment stressors interact, leading to significant two‐way and high‐order interactions? If so, what is the nature of the interactions and do these interactions vary with seasons?

## 2 Materials and Methods

### 2.1 Macroinvertebrate Sampling and Processing

**FIGURE 1:**
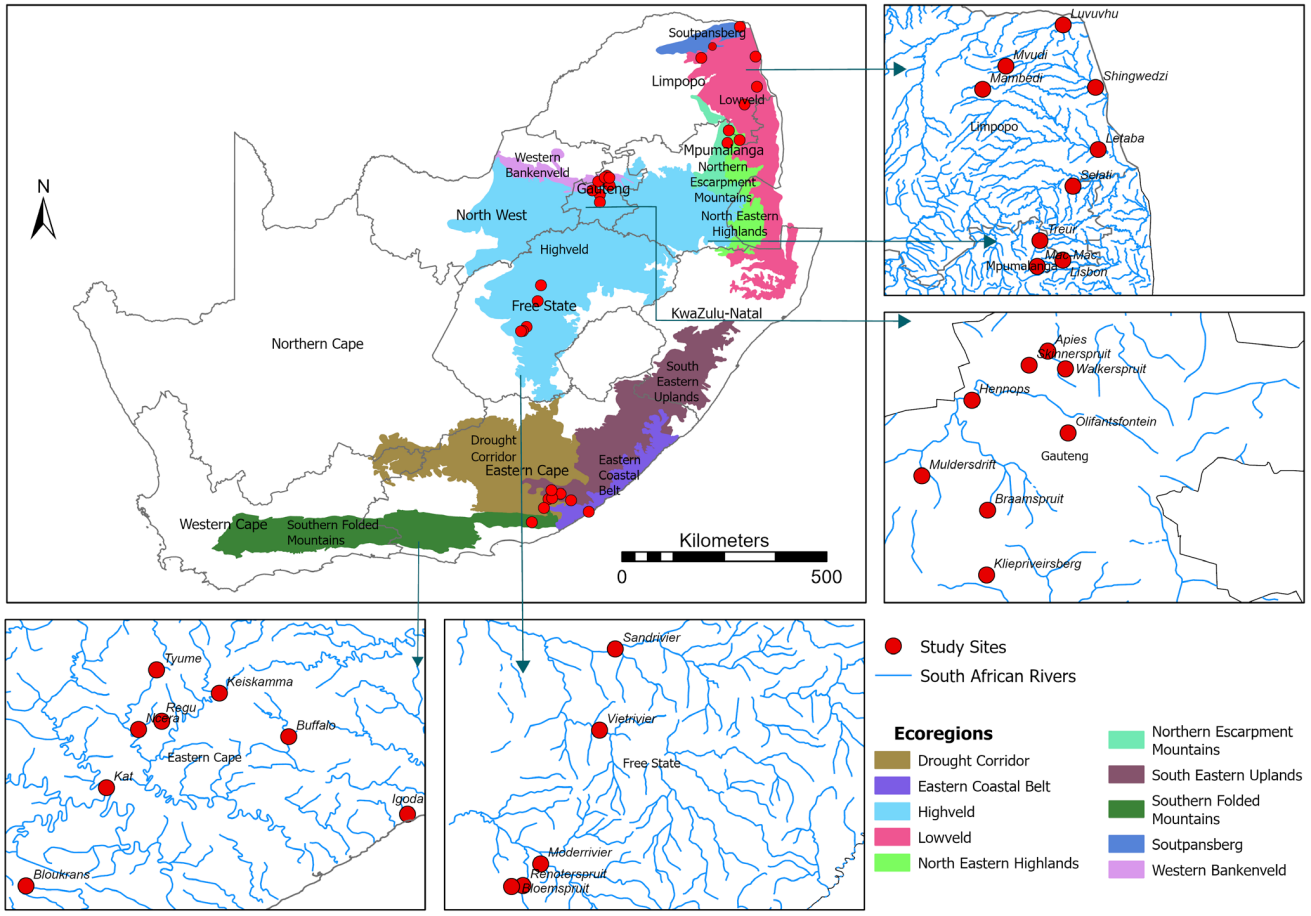
Study site locations, covering 10 ecoregions and five provinces: Eastern Cape, Free State, Gauteng, Mpumalanga and Limpopo. The arrows show the geographic location of each province in South Africa.

Macroinvertebrates were sampled from 30 first‐ and second‐order streams across a latitudinal gradient, covering five provinces and 10 ecoregions in South Africa (Figure 1), to represent diverse climatic conditions, including variation in temperature and rainfall. Ecoregions were defined as large areas that support distinct biological communities (Abell et al. 2008). Balanced representation among ecoregions was not achieved due to logical constraints and site accessibility, resulting in an unequal number of sampled sites across ecoregions. All sites were sampled in August 2023 and April 2024, representing the cool–dry and hot–wet seasons, respectively. One site, however, was not sampled during the hot–wet season due to logistical challenges. Macroinvertebrates were sampled in riffles with a Surber sampler (25 cm × 25 cm, 250 μm mesh size). All samples in each stream (*n* = 5) were preserved in 70% ethanol. Macroinvertebrates were measured and identified using regional keys to family level, except for the oligochaetes and nematodes, which were identified to subclass level. We calculated the body mass value of each individual using the allometric equation $M=a\times L^{b}$, where a and b are taxon‐specific constants derived from published length‐mass regressions (Benke et al. 1999), and L is body length (mm). Mean biomass for each taxon was then calculated by multiplying its abundance by the mean body size of its individuals.

### 2.2 Stressor Classification

In each sampling season, water quality variables (i.e., pH, conductivity (μS/cm), total dissolved solids (TDS) (mg/L) and water temperature (°C)) were measured at each of the 30 sites (*n* = 3) using a Hanna Combo Tester HI98129 (Hanna Instruments, Romania) to characterise site conditions. At each site, water samples (*n* = 3) were collected for nutrient analysis (i.e., ammonium, phosphate, and nitrate) using a Hanna multiparameter photometer (HI83300) in the laboratory.

We characterised human land cover as a percentage at each sampled site using the 2022 South African National Landcover (SANLC) data, which consists of 73 land use classes at 20 m resolution (available on https://www.dffe.gov.za/egis). To quantify the percentage, we used ArcGIS Pro to delineate a 500 m‐wide polygon on each side of the stream (Theodoropoulos et al. 2015) and extending 1 km upstream, as previous studies have shown that 1 km buffers are critical in influencing water quality (Brumberg et al. 2021; Łaszewski et al. 2022; Zhu et al. 2024). We extracted the land‐use classes within the delineated polygon and calculated the percentage cover of human land cover. Human land cover was characterised by urban and agricultural features, including settlements, plantations and farmland.

Climate data were obtained from the South African Weather Service (SAWS), recorded from 17 weather stations. We selected annual rainfall as in Carvallo et al. (2022) and mean daily maximum temperature data as representations of climate variability. We calculated climate variables for the 12 months before each sampling season, 12 months before August 2023 for the cool–dry season and 12 months before April 2024 for the hot–wet season. The August 2023 sampling period coincided with the eighth hottest year on record (1951–2024), with a mean annual temperature of 0.36°C above the 1991–2020 climatological reference period (South African Weather Service 2023). In contrast, the April 2024 sampling season coincided with the hottest year on record since 1951, with temperatures 0.9°C above the same 1991–2020 baseline. National rainfall was near normal, but the north–western interior experienced well‐below normal rainfall, while coastal and eastern regions, including Mpumalanga province, where some of the study sites are located, received well‐above normal rainfall (South African Weather Service 2024).

### 2.3 Statistical Analysis

All statistical analyses were done in R version 4.4.2 (R Core Development Team 2024). Season (hot–wet or cool–dry) was a fixed categorical variable with all other variables continuous (temperature, rainfall, ammonium, phosphate, nitrate and human land cover). We calculated seven community‐level macroinvertebrate metrics (1. taxa abundance, 2. taxa richness, 3. Ephemeroptera, Plecoptera and Trichoptera (EPT) abundance, 4. EPT richness, 5. Simpson's diversity Index (SDI), 6. mean body size (mg) and 7. community biomass (mg)) to represent macroinvertebrate community dynamics. Next, we square‐root‐transformed the relative abundance data for the macroinvertebrate communities to reduce the influence of highly abundant taxa. We applied nonmetric multidimensional scaling (NMDS) with Bray–Curtis dissimilarities to visualise variation in community composition. Absolute abundance data were retained for all subsequent statistical models. The NMDS was conducted using the meta–MDS function in the *vegan* package (Oksanen et al. 2025). We used the Shepard's plot and coefficient of determination (*R* ^2^) to evaluate the strength and reliability of the NMDS ordination.

We ran permutational multivariate analysis of variance (PERMANOVA) with 999 permutations using the ‘adonis2’ function to test if communities differed between sites and the main effects of the predictor variables. The environmental variables explaining variation were overlaid using the *envfit* function, selecting variables that have significant effects at *p* < 0.05. A post hoc exploration using Similarity percentage analysis (SIMPER) and Spearman's rank correlations was used to calculate the contribution of each taxon to the variation in macroinvertebrate composition.

We investigated multicollinearity using the variance inflation factor in the vif function from the ‘*car*’ package. The vif values for all stressor variables included in the General linear mixed effects models (GLMMs) were < 5, indicating no collinearity issues (Table S1). GLMMs were used to analyse the relationships between the response and stressor variables mentioned above using the ‘glmmTMB’ function of the ‘*glmmTMB*’ package (Aguiar Jr. et al. 2015; Brooks et al. 2017). We applied a Negative binomial for taxa and EPT abundance to account for overdispersion and a Poisson model for taxa and EPT richness. We used the Gamma distribution with a log link for Simpson's diversity index, mean body size and community biomass. A summary of the model specifications is in Table S3. Model diagnostic checks were performed using the *DHARMa* package for overdispersion and outliers. We looked at all possible predictor combinations of our 6 continuous variables (human land cover, ammonium, phosphate, nitrate, temperature and rainfall) and 1 categorical variable (season [hot–wet or cool–dry]) and picked the best model for each response variable based on Akaike information criterion (AIC_c_) using the dredge function in the *MuMIn* package (Bartoń 2026; Table S2). AIC_c_ selects the most parsimonious model and provides the balance between overfitting and underfitting (Altwegg et al. 2006). We retained models with the lowest AIC_c_ values as the best predictors of responses and also considered models within two units of the top‐ranked model (ΔAIC_c_ ≤ 2) for inference. If the best‐fit and ΔAIC_c_ ≤ 2 models were statistically significant (*p* < 0.05), we interpreted and reported all such models. Random effects for ecoregion and site were considered to account for the hierarchical sampling design. Including the random effects also accounted for the five sites that had incomplete nutrient enrichment data for one season (due to missing samples and instrument failure) and were, therefore, excluded from the analysis for that season only. The resulting models were response ~ predictor (1|Site) + (1|Ecoregion) for the main effects, response ~ predictor 1 × predictor 2 (1|Site) + (1|Ecoregion) for the two‐way interaction, and response ~ predictor 1 × predictor 2 × predictor 3 (1|Site) + (1|Ecoregion) for the three‐way interaction. Season was further added to the main and interaction models to test the influence of seasonal differences on stressor interaction.

We computed marginal and conditional *R* ^2^ values (Nakagawa and Schielzeth 2012) for each mixed‐effects model. To account for zero variance associated with random effects, which can inflate AIC_c_, lead to singular fits and unreliable estimates of marginal and conditional *R* ^2^, we used the VarCorr() function in the *lme4* package to estimate the variance explained by each random effect (Bates et al. 2015). If only one random effect explained zero variance, it was removed to simplify the model. If both random effects explained zero variance, they were removed from the model following the approach of Ehlman et al. (2020), leading to a simplified general linear model (GLM). The specific random effects included in the modelling for each response variable are summarised in Table S3. Considering that AIC_c_ models need to be fitted using maximum likelihood (ML) to be comparable across different random effect structures (Pinheiro and Bates 2000), we did not make any additional adjustments in our analysis, as the glmmTMB fits by ML by default. As described by Brooks et al. (2017), the log‐likelihoods are calculated consistently in *glmmTMB*, which allows comparison of likelihood‐based model comparison metrics to remain statistically valid even when random effect structure differs between models. Model estimates are reported as the Beta (*β*) coefficient from the GLMMs, representing the direction of each stressor's variable and interactions with the response variable for the interpretation of the direction of interaction terms.

## 3 Results

### 3.1 Environmental Characteristics of Streams

Environmental conditions varied across streams and seasons, reflecting site‐specific characteristics (Table 1). Mean water temperature was 16°C (range 11°C–23°C) in the cold–dry season and 20°C (range 16°C–32°C) in the hot–wet season. Water pH was generally neutral to alkaline, ranging from 7.0 to 8.5 in the cool–dry season and from 6.8 to 7.9 in the hot–wet season. Mean total dissolved solids (TDS) were 320 mg/L in the cool–dry season and 255 mg/L in the hot–wet season. Conductivity ranged from 29.9–2414.3 μS/cm in the cool–dry season and 23.3–2237.7 μS/cm in the hot–wet season. The percentage of human land cover ranged from 0% to 63%, with a mean ± standard deviation of 11% ± 14%, suggesting considerable variation in human land cover across the sampled sites. These environmental variables provide important context for interpreting how abiotic conditions influence stream macroinvertebrate communities. Additional information about the environmental characteristics of the streams is provided in Table S4.

**TABLE 1:** Variation in environmental variables over the two sampling seasons in the 30 sampled sites given as mean ± standard deviation (with range in brackets).

Environmental variables	Season	
	Cool–dry	Hot–wet
pH	7.8 ± 0.5 (7–8.5)	7.5 ± 0.4 (6.8–8.0)
Water temperature	16.1 ± 2.7 (10.8–22.7) °C	19.6 ± 3.5 (15.8–31.6) °C
Total dissolved solids	320.3 ± 295.0 (0–1027.3) mg/L	255.1 ± 257.3 (1.29–1063.7)
Conductivity	702.8 ± 620.0 (29.9–2414.3)	687.4 ± 631.9 (23.31–2237.7)

### 3.2 Community Composition

**FIGURE 2:**
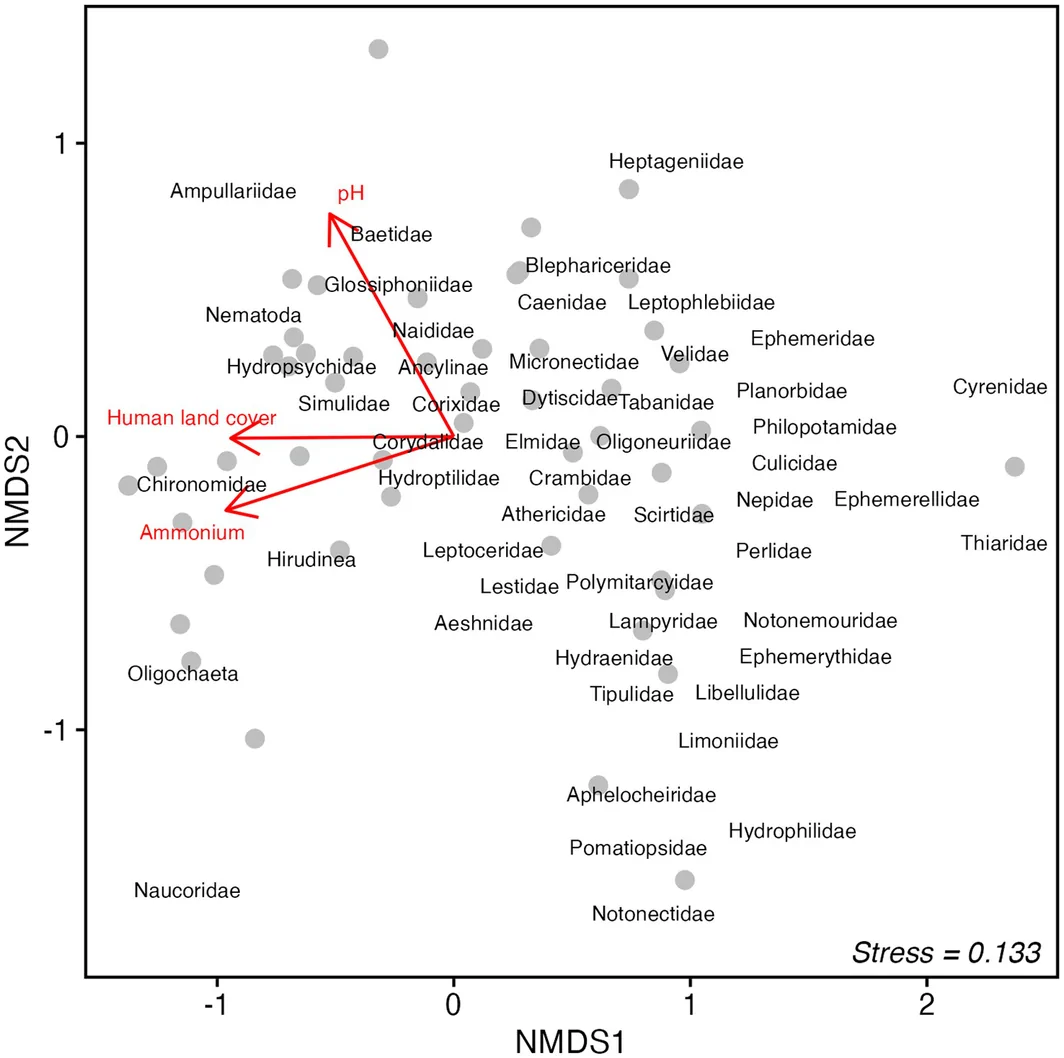
Non‐metric multidimensional scaling (NMDS) ordination based on Bray–Curtis dissimilarity. Envifit vectors show the environmental variables associated with community abundance (*p* < 0.05).

A total of 10,816 macroinvertebrates belonging to 59 families were identified, with Chironomidae, Simuliidae, Baetidae, Caenidae and Hydropsychidae as the dominant groups. The NMDS plot had a stress value of 0.133, indicating good and reliable representation of the abundance data, and the Shepard diagram confirmed this pattern (*R* ^2^ = 0.902; Figure S1). Our analysis showed that pH, ammonium, and human land cover were the variables strongly associated with the variation in community composition (Figure 2). PERMANOVA confirmed this pattern for human land cover (PERMANOVA, pseudo‐*F* = 2.872, *R* ^2^ = 0.052, *p* = 0.002) and pH (PERMANOVA, pseudo‐*F* = 3.117, *R* ^2^ = 0.057, *p* < 0.001). The full PERMANOVA results are summarised in Table S5.

### 3.3 General Linear Models

**FIGURE 3:**
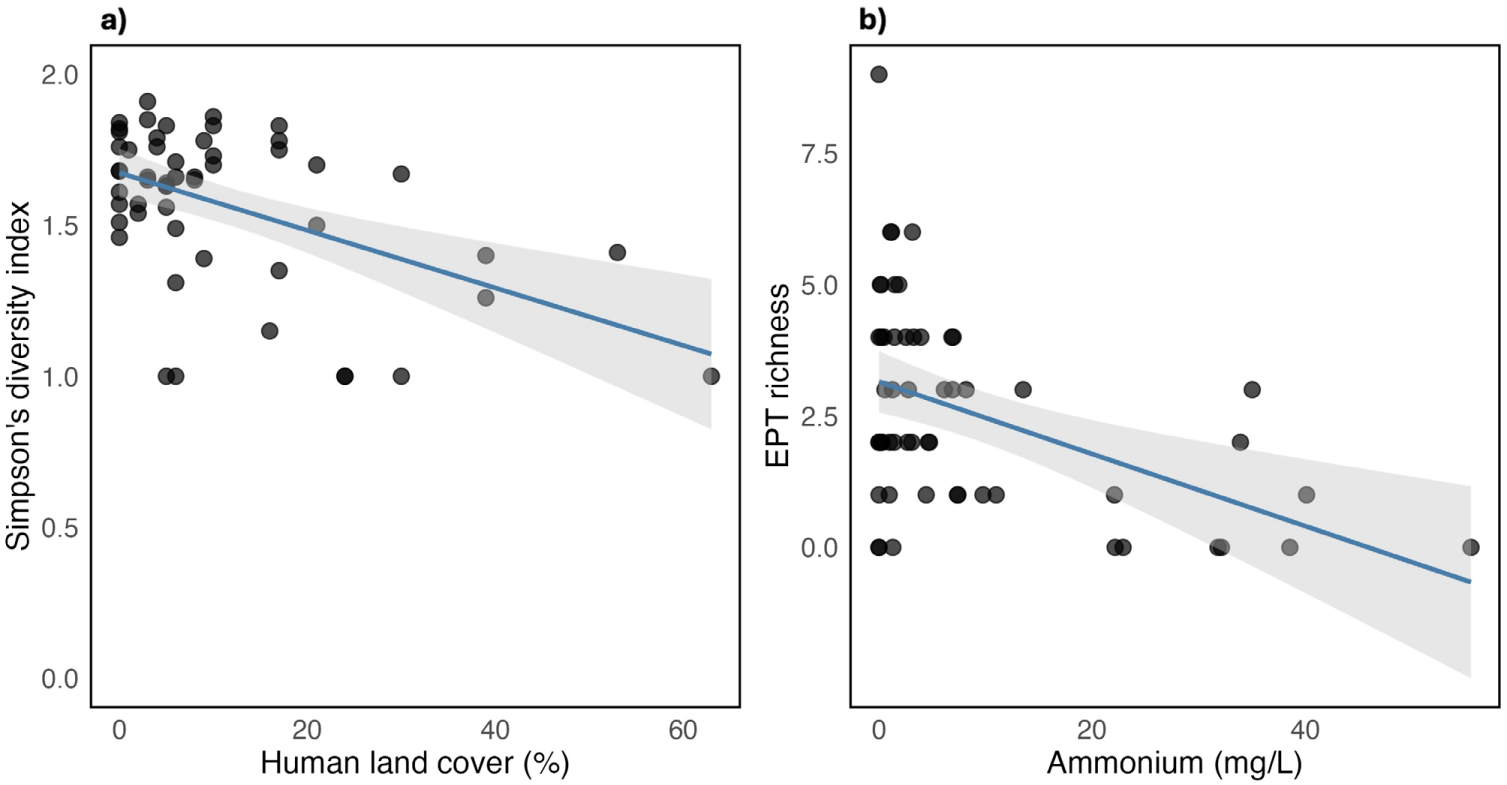
Main effects of (a) % of human land cover on Simpson's diversity index; and main effects of ammonium on (b) EPT richness. The solid blue line and shaded grey area are predicting the fitting and 95% confidence intervals from the GLMMs.

Simpson's diversity index declined significantly with increasing human land cover (AIC_c_ = 7.3, *β* = −0.007 ± 0.001, *p* < 0.001; Figure 3a), while EPT richness declined with increasing ammonium concentration (AIC_c_ = 200.2, *β* = −0.038 ± 0.012, *p* = 0.002; Figure 3b). Mean biomass was best explained by temperature; however, this was not statistically significant (*p* > 0.05). The human land cover × temperature and human land cover × ammonium × temperature were within two AIC_c_ units (ΔAIC_c_ ≤ 1.9). However, within these models, only the main effects of ammonium were statistically significant (*β* = 1.024 ± 0.507, *p* < 0.044; Figure S2), implying no evidence for interactive effects; instead, mean biomass increased with ammonium concentration. Post‐hoc analysis using Spearman rank correlations revealed that this positive effect was primarily driven by pollution‐tolerant taxa such as Chironomidae, Micronectidae and Corixidae (Table S6).

**FIGURE 4:**
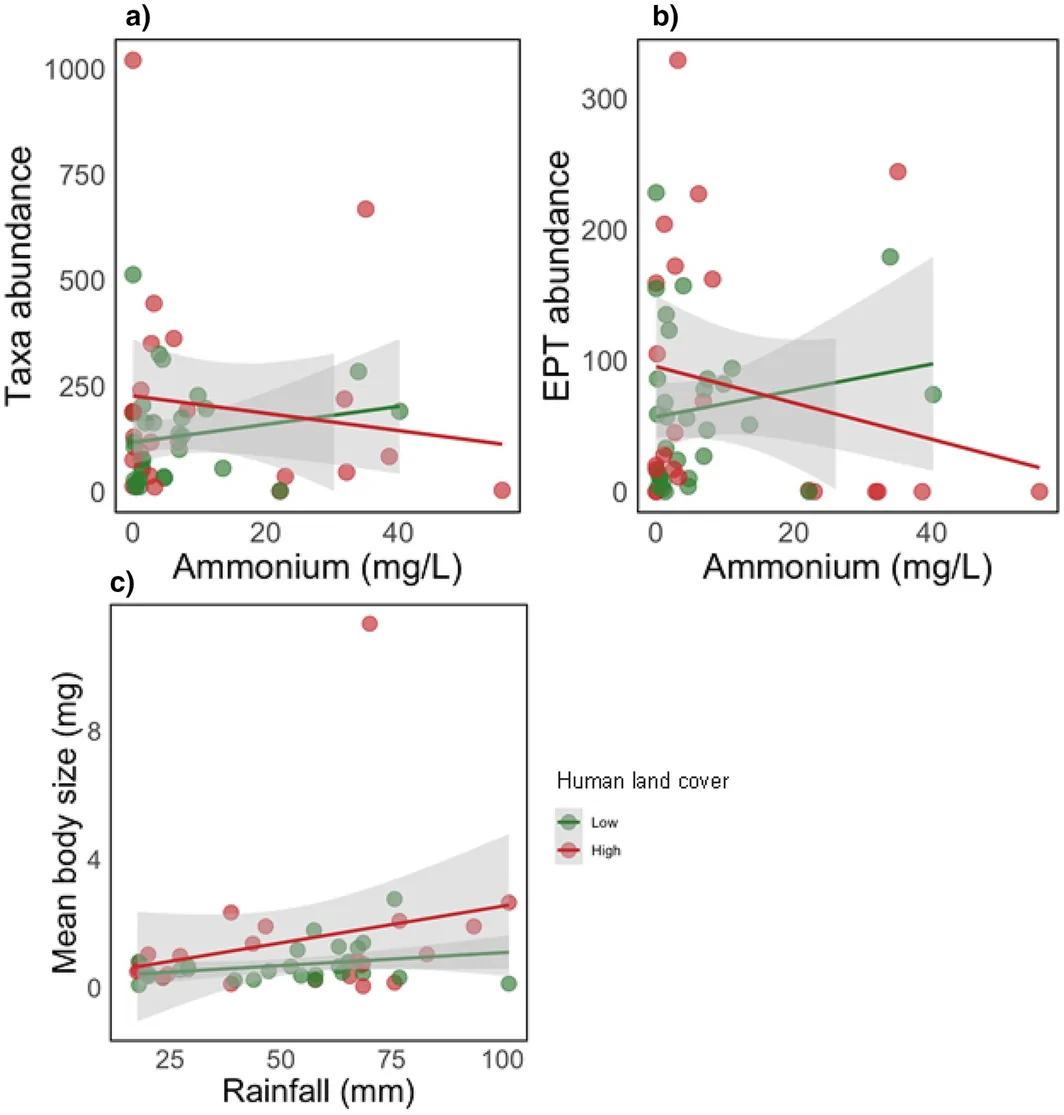
Interactive effects of (a) ammonium and human land cover on taxa abundance, (b) ammonium and human land cover on EPT abundance, (c) rainfall and human land use on mean body size. Here, rainfall and human land cover are categorised into low and high to simplify visualisation trends, but in the models, we used continuous data.

The interactive effects of human land cover and ammonium were the best‐fit model for taxa abundance (AIC_c_ = 660.7, *β* = −0.003 ± 0.001, *p* = 0.004; Figure 4a) and EPT abundance (AIC_c_ = 545.4, *β* = −0.038 ± 0.012, *p* < 0.002; Figure 4b) indicating that the effects of ammonium on these two responses depended on the level of human land cover. SIMPER showed that the decline in taxa abundance with high ammonium and high human land cover was driven by the decline in the most abundant taxa (i.e., Chironomidae, Simuliidae, Caenidae, and Baetidae; Table S7).

The intercept had the lowest AIC_c_ for taxa richness, suggesting limited evidence for effects of the stressors on taxa richness (Table S2). Mean body size was best predicted by human land cover × rainfall (AIC_c_ = 95.9, *β* = 0.001 ± 0.001, *p* = 0.01; Figure 4c). The ammonium × rainfall × season model was within 2 AIC_c_ units and statistically significant (ΔAIC_c_ = 1.6; *β* = −0.003 ± 0.001, *p* = 0.002; Figure S3), suggesting that interactive effects of ammonium and rainfall varied with season on mean body size. *DHARMa* identified outliers for this interactive model on mean body size; however, excluding the outliers did not change the statistical significance of the model.

## 4 Discussion

Freshwater biodiversity is increasingly threatened by the combined effects of multiple stressors, and understanding their interactions is crucial in the context of global environmental change (Kefford et al. 2023). Our study demonstrates that both the single and interactive effects of multiple stressors associated with climate and anthropogenic stressors play an important role in driving macroinvertebrate community dynamics in freshwater ecosystems in South Africa. Ammonium was the most pervasive stressor with negative consequences for metrics for both macroinvertebrate abundance and richness. We found independent effects of ammonium on EPT richness and mean biomass, and of human cover on Simpson's diversity index, but no independent effects of climate stressors. We found no high‐order interactions; instead, all interactions were two‐way. For instance, human land cover determined the scale of the effects of ammonium on invertebrate abundance.

### 4.1 Independent Effects

Modification of natural land use to agricultural and urban land use is strongly associated with the homogenisation of macroinvertebrate taxa and the loss of diversity (Lu et al. 2022; Mahmud et al. 2024; Rumschlag et al. 2023). In our study, the percentage of human land cover translated into clear negative effects on Simpson's diversity index. This can be attributed to increased runoff and nutrient inputs, including the deposition of contaminants from urban and agricultural streams (Chen et al. 2025; Wang et al. 2025). Our study only quantified land use within 1 km from the sampled points, yet considerable effects on Simpson's diversity were still evident. This suggests that even moderate levels of human land cover are sufficient to trigger changes in diversity in freshwater ecosystems, consistent with previous research that macroinvertebrates show greater sensitivity to urban and agricultural land use, with ecological thresholds of 2%–10% and 15%–40%, respectively, with pronounced responses within 1–5 km (Xie et al. 2025). Therefore, managing land use change within freshwaters remains an important conservation effort under multiple stressors.

We found negative effects of ammonium on EPT richness, however, it is important to note that ammonium in this study reflects conditions at the time of sampling rather than long–term patterns, which limits generalising short‐term effects to long‐term patterns. Nevertheless, these short‐term effects provide another important mechanism for why responses to stressors involving nutrient enrichment may vary between short‐term and long‐term contexts and may account for the inconsistencies observed across ecological studies. The negative effects of nutrient enrichment on macroinvertebrate communities are a common and widely reported trend in freshwater studies (e.g., Lin et al. 2021; Birk et al. 2020; Lourenço et al. 2023). Our findings are therefore consistent with numerous studies (e.g., Let et al. 2022; Nessel et al. 2023; Barasa et al. 2025) that have found nutrient enrichment to be strongly associated with declines in EPT abundance and richness. Potential mechanisms for such effects include nutrient enrichment leading to shifts from diatom‐dominated assemblages to cyanobacteria‐dominated communities. This shift disrupts consumer‐resource interactions, and reduces dissolved oxygen. Collectively, these processes alter stream conditions and changes in community composition (Elser et al. 2007; Isbell et al. 2013). The EPT taxa are highly sensitive to low oxygen and increased organic loading, so such nutrient‐driven changes lead to reduced survival and reduce the ability of macroinvertebrates to coexist, ultimately lowering EPT richness, instead, promoting a stable, low‐diversity state (Downing et al. 2012). In contrast, we found positive effects of ammonium on mean biomass, suggesting that the effects of nutrient enrichment on freshwater ecosystems differ with ecological responses. However, post‐hoc analysis revealed that this increase was driven by pollution‐tolerant taxa such as Chironomidae and Micronectidae, consistent with previous research (e.g., Cortelezzi et al. 2015; Neijnens et al. 2024), which demonstrated that in freshwater ecosystems, nutrient enrichment leads to increased abundance of stress‐tolerant species. The dominance of pollution‐tolerant taxa likely reflects ecological degradation and has potential negative effects on freshwater ecosystem functioning and processes.

### 4.2 Stressor Interaction

Total taxa and EPT abundance were reduced under conditions of both high concentrations of ammonium and a high percentage of human land cover, suggesting that these two stressors exacerbate each other's negative effects. Two potential interacting mechanisms may explain these findings. First, land use changes degrade freshwater habitats through the removal of riparian vegetation (Fierro et al. 2017; Zermeño‐Hernández et al. 2020), the sedimentation of aquatic ecosystems (Liu et al. 2017), and the homogenisation of stream substrates (Camana et al. 2024), and alterations in flow (Cooper et al. 2013; Kayitesi et al. 2022). Second, natural land use is known to modulate nutrient input, support freshwater biodiversity (Sargac et al. 2021), and mitigate diffuse pollution (Cole et al. 2020). By implication, modification of natural land use diminishes these important functions, further degrading freshwater ecosystems, leading to reduced abundance of macroinvertebrate communities. The decline in EPT abundance is consistent with a previous study showing that nutrient enrichment and human land use interact and disproportionately affect sensitive taxa (Lee et al. 2020; Gücker et al. 2024; Chen et al. 2025). These findings highlight the importance of land use management and reductions in nutrient input to manage freshwater ecosystems in the face of growing multiple stressors in freshwater ecosystems. These two stressors are intertwined, as human land cover in the form of agricultural and urban land cover contributes to ammonium concentrations in riverine ecosystems. Understanding their interaction is crucial in the management of freshwater ecosystems as it allows us to account for ammonium associated with non‐point source (i.e., agricultural and urban land cover) and from other sources, which can be disregarded if we consider one stressor alone. Future studies can employ methods that allow accurate disentanglement of different land use patterns and nutrient enrichment effects to advance our understanding of the interactive effects of point‐source and non‐point source pollution.

Both the human land cover × rainfall and ammonium × rainfall × season interactions showed positive effects on mean body size, indicating that rainfall is an important stressor in modulating the effects. Human land use and nutrient input are important pathways for food resources for macroinvertebrates in freshwater ecosystems (Wang et al. 2025). However, excess nutrient input can have negative effects, leading to simplification of community structure (Xie et al. 2024), and our findings show that rainfall and hot–wet seasons can buffer against this dynamic. The observed increase in mean body size in our study may be explained by higher rainfall and a hot–wet season, flushing excess nutrients, mitigating the accumulation of excess nutrients, while maintaining conditions that support larger body size in macroinvertebrate communities. These positive findings contrast with previous research reporting negative effects of higher nutrients and higher runoff leading to declines in macroinvertebrates (Mantyka‐Pringle et al. 2014). However, Mantyka‐Pringle et al. (2014) focused on macroinvertebrate abundance, suggesting that macroinvertebrate metrics can respond differently to the same stressors.

Our findings on the interactive effects of climate, land use and nutrient enrichment provided evidence for both independent effects and interactive effects, contrasting previous research, which has shown that non‐interactive effects are more common in field‐based than in experimental studies in freshwater ecosystems (Gutiérrez‐Cánovas et al. 2022; Lourenço et al. 2025). We highlighted that nutrient enrichment, specifically ammonium concentration, contributed significantly more to macroinvertebrate responses than did human land cover and climate stressors. We found no effects of temperature across all seven responses, despite sampling in seasons that coincided with the hottest years on record. This implies that local stressors are the major drivers of macroinvertebrate dynamics in freshwater ecosystems.

### 4.3 Scale‐Dependent Ecological Responses to Interacting Stressors

By revisiting a subset of the streams originally surveyed by Jackson et al. (2020) in 2014–2015, we were able to evaluate the temporal consistency of ecological responses to climate and land‐use stressors almost a decade later. Although the two studies focused on different levels of biological organisation, food‐web structure quantified using stable isotopes in the earlier study and community composition and body‐size distributions in the present study, both examined the ecological consequences of the same overarching environmental drivers. This comparison therefore provides a rare opportunity to assess whether ecological responses to multiple stressors are consistent across organisational levels and through time. Jackson et al. (2020) reported significant independent effects of both land use and climate stressors on food‐web structure, whereas our analyses revealed no independent effects of climate stressors and only limited independent effects of land use, restricted to Simpson's diversity. Despite these differences, both studies identified non‐additive interactions among stressors, highlighting the importance of considering stressor combinations rather than individual drivers in isolation. Notably, however, the direction of these interactions differed, with Jackson et al. (2020) observing negative interactive effects on isotopic uniqueness, whereas we found positive interactive effects on mean body size. Such contrasting outcomes suggest that responses to multiple stressors are highly dependent on the ecological attribute being measured and may vary among levels of biological organisation.

Additional differences emerged in the factors regulating community structure, where Jackson et al. (2020) observed that seasonal variation was a dominant driver of invertebrate abundance, whereas in the present study taxa abundance was primarily explained by interactions between land use and ammonium concentrations. Furthermore, seasonal variability modified the influence of human land cover and nutrient enrichment on mean body size, indicating that temporal environmental fluctuations can alter the strength and direction of anthropogenic stressor effects. These findings demonstrate that ecological responses observed at the food‐web level do not necessarily translate to community‐level or trait‐based responses, even within the same ecosystems. Our findings support the growing recognition that the effects of multiple anthropogenic stressors are rarely universal, instead varying across ecological scales, levels of biological organisation, and environmental contexts. Therefore, caution is warranted when extrapolating findings from a single level of biological organisation to ecosystem‐wide responses. Conservation and management strategies should therefore adopt a multi‐level ecological perspective that explicitly recognises the potential for divergent responses among species, communities, and food webs under interacting environmental stressors.

### 4.4 Limitations and Future Research

The results observed in this study may be limited by how the stressors were quantified. Nutrient enrichment stressors were assessed from samples collected during the study sampling period, rather than long‐term monitoring data, as was available for temperature and rainfall, with two sites missing samples and three additional sites excluded due to instrument failure. As such, they may not fully capture temporal variability. Nonetheless, they remain highly informative as they capture short‐term nutrient conditions affecting macroinvertebrate communities and therefore provide insights into immediate ecological responses to nutrient exposure even under ongoing long‐term climatic changes. Future research could examine the patterns using long‐term data and consider other metrics such as functional traits, population dynamics and species‐specific responses. The results of this study may also be limited by the spatial coverage of the classified land use. Future studies can use multiple distance ranges to examine the degree to which effects vary with the increasing extent of classified land use.

### 4.5 Conclusion and Management Implications

Managing freshwater ecosystems in the face of global change requires a clear understanding of how multiple stressors shape biological communities (Emmons et al. 2025; Kong et al. 2025; Spiller et al. 2025). Considering the important role of macroinvertebrate communities on stream ecosystem functioning and processes, this study advances our understanding of their responses to multiple stressors, which is important for freshwater conservation. Our study demonstrates that ecological responses in natural ecosystems were not driven by single effects alone; rather, stressors interacted, producing context‐dependent biological outcomes. Importantly, climate‐related stressors had no main effects but interacted to shape responses, while anthropogenic stressors showed independent effects. These findings highlight the combined and context‐dependent effects of multiple stressors in freshwater ecosystems and emphasise that management decisions based on individual stressors might not be enough to conserve freshwater ecosystems under global change. Here, we show that management strategies should prioritise reductions in nutrient input and improve catchment land management, while also recognising context‐dependencies in stressor interactions, considering the antagonistic responses.

## Author Contributions

**Buntu Fanteso:** conceptualization (lead), data curation (lead), formal analysis (lead), funding acquisition (lead), investigation (lead), methodology (lead), project administration (lead), validation (lead), visualization (lead), writing – original draft (lead), writing – review and editing (lead). **Tatenda Dalu:** conceptualization (equal), data curation (equal), funding acquisition (equal), resources (equal), supervision (supporting), writing – review and editing (equal). **Ryan Wasserman:** resources (equal), writing – review and editing (equal). **Wilbert Kadye:** funding acquisition (equal), resources (equal), writing – review and editing (equal). **Pule Mpopetsi:** data curation (supporting), writing – review and editing (equal). **Michelle Jackson:** conceptualization (equal), funding acquisition (equal), supervision (lead), writing – review and editing (equal).

## Conflicts of Interest

The authors declare no conflicts of interest.

## Supporting information


**Figure S1:** Shepard plot on the observed dissimilarity vs. ordination distance showing how well the NMDS plot represents the macroinvertebrate community abundance.
**Table S1:** Variance inflation factor (VIF) metrics of the predictor and water quality variables. VIF = 1 shows no multicollinearity; 1 < VIF < 5 indicates little collinearity, but no further investigation is needed and VIF ≥ 5 shows high collinearity and warrants investigation.
**Table S2:** Best fit model selection for the effects of climate, human land cover, season and nutrient enrichment on different ecological responses. The best model for each response and the models within two AIC units of the best‐fit model are highlighted in yellow. The Conditional *R* ^2^ and Marginal *R* ^2^ are left blank for candidate models where they explained zero variance.
**Table S3:** Best‐fit models, distribution and random effect choices for each of the seven response variables.
**Table S4:** Site information for the 30 sites.
**Table S5:**: Randomisation test of the main effects.
**Figure S2:** Ammonium effects on mean biomass.
**Figure S3:** Ammonium, rainfall and season interaction on mean body size.
**Table S6:** Taxa contributing to biomass increase with ammonium.
**Table S7:** Taxa contribution to the interactive effects of ammonium and human land cover.

## Data Availability

The data supporting the study is made available on figshare: https://figshare.com/s/6afd5485ca7d035f9050.
