# Supplementary material for: The Individual and Interactive Effects of Climate, Land Use and Nutrient Enrichment on Macroinvertebrate Dynamics in South African Rivers
Source: Ecol Evol. 2026 Aug 31;16(9):e74252. doi: 10.1002/ece3.74252 (PMC13530861; doi:10.1002/ece3.74252)
Supplement: Supplementary file 1 [file ECE3-16-e74252-s001.docx]

**Figure S1:** Shepard plot on the observed dissimilarity vs ordination distance showing how well the NMDS plot represents the macroinvertebrate community abundance.

**Table S1:** Variance Inflation Factor (VIF) metrics of the predictor and water quality variables. VIF = 1 shows no multicollinearity; 1 < VIF < 5 indicates little collinearity, but no further investigation is needed and VIF ≥ 5 shows high collinearity and warrants investigation.

| Variable | vif |
| --- | --- |
| Human land cover | 1.88 |
| Ammonium | 1.62 |
| Phosphate | 1.27 |
| Nitrate | 1.85 |
| Temperature | 2.33 |
| Rainfall | 2.68 |
| Water temperature | 3.15 |
| Conductivity | 6.08 |
| Season | 3.04 |
| TDS | 5.30 |
| pH | 1.91 |
| Depth | 1.43 |
| Width | 1.76 |

**Table S2:** Best fit model selection for the effects of climate, human land cover, season and nutrient enrichment on different ecological responses. The best model for each response and the models within two AIC units of the best-fit model are highlighted in yellow. The Conditional R^2^ and Marginal R^2^ are left blank for candidate models where they explained zero variance.

| Response | Predictors | AICc | Conditional R^2^ | Marginal R^2^ |
| --- | --- | --- | --- | --- |
| Taxa abundance | Intercept | 662.1 | 0.388 | 0.000 |
|  | Human land cover | 664.5 | 0.365 | 0.002 |
|  | Phosphate | 663.7 | 0.350 | 0.023 |
|  | Nitrate | 664.5 | 0.388 | 0.000 |
|  | Ammonium | 664.5 | 0.396 | 0.002 |
|  | Temperature | 664.5 | 0.372 | 0.002 |
|  | Rainfall | 664.1 | 0.388 | 0.008 |
|  | Season | 663.5 | 0.387 | 0.020 |
|  | Ammonium * Season | 667.6 | 0.351 | 0.048 |
|  | Phosphate * Season | 667.7 | 0.312 | 0.055 |
|  | Nitrate* Season | 667.8 | 0.368 | 0.043 |
|  | Temperature * Season | 667.0 | 0.412 | 0.057 |
|  | Human land cover * Season | 666.4 | 0.429 | 0.102 |
|  | Rainfall * Season | 667.3 | 0.364 | 0.060 |
|  | Human land cover * Phosphate | 668.4 | 0.292 | 0.045 |
|  | Human land cover * Nitrate | 667.3 | 0.261 | 0.098 |
|  | Ammonium * Nitrate | 669.2 | 0.378 | 0.013 |
|  | Ammonium * Phosphate | 667.4 | 0.315 | 0.045 |
|  | Phosphate * Nitrate | 668.8 | 0.346 | 0.024 |
|  | Human land cover * Ammonium | 660.7 | 0.449 | 0.232 |
|  | Human land cover * Temperature | 666.5 | 0.422 | 0.177 |
|  | Human land cover * Rainfall | 669.3 | 0.375 | 0.011 |
|  | Phosphate * Rainfall | 668.4 | 0.325 | 0.033 |
|  | Nitrate * Temperature | 669.7 | 0.375 | 0.002 |
|  | Nitrate * Rainfall | 669.1 | 0.390 | 0.015 |
|  | Ammonium * Temperature | 669.4 | 0.387 | 0.013 |
|  | Ammonium * Rainfall | 666.7 | 0.265 | 0.087 |
|  | Human land cover * Phosphate * Temperature | 666.7 |  |  |
|  | Human land cover * Phosphate * Rainfall | 674.4 | 0.295 | 0.224 |
|  | Human land cover * Nitrate * Temperature | 666.8 |  |  |
|  | Human land cover * Nitrate * Rainfall | 677.3 | 0.341 | 0.130 |
|  | Human land cover * Ammonium * Temperature | 667.7 | 0.522 | 0.332 |
|  | Human land cover * Ammonium * Rainfall | 665.7 | 0.603 | 0.485 |
|  | Ammonium * Human land cover * Season | 668.1 | 0.488 | 0.342 |
|  | Ammonium * Rainfall * Season | 676.2 | 0.332 | 0.145 |
|  | Ammonium * Phosphate * Season | 672.9 | 0.410 | 0.192 |
|  | Ammonium * Phosphate * Nitrate | 675.8 | 0.414 | 0.125 |
|  | Ammonium * Nitrate * Season | 677.0 | 0.262 | 0.179 |
|  | Phosphate * Temperature * Season | 677.9 | 0.403 | 0.089 |
|  | Phosphate * Human land cover * Season | 670.5 | 0.368 | 0.276 |
|  | Phosphate * Rainfall * Season | 678.4 | 0.331 | 0.081 |
|  | Temperature * Rainfall * Season | 676.4 | 0.394 | 0.144 |
|  | Temperature * Human land cover * Season | 672.3 | 0.600 | 0.261 |
|  | Human land cover * Season * Rainfall | 676.5 | 0.310 | 0.140 |
| Taxa richness | Intercept | 271 | 0.680 | 0.000 |
|  | Human land cover | 271.1 | 0.660 | 0.067 |
|  | Phosphate | 273.3 | 0.680 | 0.000 |
|  | Nitrate | 272.5 | 0.675 | 0.009 |
|  | Ammonium | 272.4 | 0.653 | 0.018 |
|  | Temperature | 272.4 | 0.695 | 0.029 |
|  | Rainfall | 273.3 | 0.680 | 0.000 |
|  | Season | 272.4 | 0.681 | 0.007 |
|  | Ammonium * Season | 274.5 | 0.648 | 0.061 |
|  | Phosphate * Season | 276.9 | 0.677 | 0.011 |
|  | Nitrate* Season | 274.7 | 0.673 | 0.036 |
|  | Temperature * Season | 275.4 | 0.699 | 0.037 |
|  | Human land cover * Season | 274.5 | 0.661 | 0.085 |
|  | Rainfall * Season | 273.9 | 0.662 | 0.06 |
|  | Human land cover * Phosphate | 273.2 | 0.659 | 0.098 |
|  | Human land cover * Nitrate | 274.2 | 0.657 | 0.114 |
|  | Ammonium * Nitrate | 275.4 | 0.645 | 0.066 |
|  | Ammonium * Phosphate | 272.6 | 0.625 | 0.124 |
|  | Nitrate * Phosphate | 276.2 | 0.659 | 0.032 |
|  | Human land cover * Ammonium | 274.6 | 0.666 | 0.104 |
|  | Human land cover * Temperature | 275.6 | 0.678 | 0.079 |
|  | Human land cover * Rainfall | 272.5 | 0.679 | 0.108 |
|  | Phosphate * Rainfall | 276.5 | 0.669 | 0.017 |
|  | Nitrate * Temperature | 275.8 | 0.686 | 0.051 |
|  | Nitrate * Rainfall | 275.1 | 0.671 | 0.055 |
|  | Ammonium * Temperature | 276.7 | 0.670 | 0.039 |
|  | Ammonium * Rainfall | 274.0 | 0.637 | 0.102 |
|  | Human land cover * Phosphate * Temperature | 282.4 | 0.681 | 0.112 |
|  | Human land cover * Phosphate * Rainfall | 276.9 | 0.663 | 0.146 |
|  | Human land cover * Nitrate * Temperature | 282.1 | 0.652 | 0.185 |
|  | Human land cover * Nitrate * Rainfall | 278.7 | 0.681 | 0.208 |
|  | Human land cover * Ammonium * Temperature | 278.1 | 0.685 | 0.169 |
|  | Ammonium * Human land cover * Season | 279.4 | 0.679 | 0.220 |
|  | Ammonium * Rainfall * Season | 276.8 | 0.674 | 0.308 |
|  | Ammonium * Phosphate * Nitrate | 275.6 | 0.636 | 0.222 |
|  | Human land cover * Temperature | 275.6 | 0.678 | 0.079 |
|  | Phosphate * Human land cover * Season | 280.7 | 0.649 | 0.143 |
|  | Human land cover * Ammonium * Temperature | 278.1 | 0.685 | 0.169 |
|  | Human land cover * Ammonium * Rainfall | 273.3 | 0.697 | 0.227 |
|  | Ammonium * Phosphate * Nitrate | 275.6 | 0.636 | 0.222 |
|  | Ammonium * Nitrate * Season | 283.6 | 0.650 | 0.095 |
|  | Phosphate * Temperature * Season | 285.4 | 0.697 | 0.038 |
|  | Phosphate * Human land cover * Season | 280.7 | 0.649 | 0.143 |
|  | Phosphate * Rainfall * Season | 281.6 | 0.649 | 0.112 |
|  | Temperature * Rainfall * Season | 283.9 | 0.673 | 0.096 |
|  | Temperature * Human land cover * Season | 283.6 | 0.683 | 0.102 |
|  | Human land cover * Season * Rainfall | 280.4 | 0.664 | 0.189 |
| EPT abundance | Intercept | 547.7 |  |  |
|  | Human land cover | 550.4 | 0.452 | 0.256 |
|  | Phosphate | 552.2 |  |  |
|  | Nitrate | 549.7 |  |  |
|  | Ammonium | 549.7 |  |  |
|  | Temperature | 549.6 |  |  |
|  | Rainfall | 549.9 |  |  |
|  | Season | 549.5 |  |  |
|  | Ammonium * Season | 559.3 |  |  |
|  | Phosphate * Season | 551.9 |  |  |
|  | Nitrate * Season | 553.3 |  |  |
|  | Temperature * Season | 553.8 |  |  |
|  | Human land cover * Season | 553.7 | 0.574 | 0.344 |
|  | Rainfall * Season | 550.0 |  |  |
|  | Human land cover * Phosphate | 552.7 |  |  |
|  | Human land cover * Nitrate | 553.2 | 0.698 | 0.396 |
|  | Ammonium * Nitrate | 553.9 |  |  |
|  | Ammonium * Phosphate | 551.7 |  |  |
|  | Nitrate * Phosphate | 552.1 |  |  |
|  | Human land cover * Ammonium | 545.4 | 0.774 | 0.545 |
|  | Human land cover * Temperature | 553.5 | 0.593 | 0.365 |
|  | Human land cover * Rainfall | 555.1 | 0.682 | 0.266 |
|  | Phosphate * Rainfall | 552.2 |  |  |
|  | Nitrate * Temperature | 554.1 |  |  |
|  | Nitrate * Rainfall | 553.9 |  |  |
|  | Ammonium * Temperature | 553.9 | 0.581 | 0.224 |
|  | Ammonium * Rainfall | 553.3 |  |  |
|  | Human land cover * Phosphate * Temperature | 553.3 |  |  |
|  | Human land cover * Phosphate * Rainfall | 556.5 |  |  |
|  | Human land cover * Nitrate * Temperature | 552.6 |  |  |
|  | Human land cover * Nitrate * Rainfall | 560.7 | 0.698 | 0.510 |
|  | Human land cover * Ammonium * Temperature | 553.7 | 0.785 | 0.653 |
|  | Ammonium * Human land cover * Season | 550.8 | 0.833 | 0.657 |
|  | Ammonium * Rainfall * Season | 556.1 | 0.824 | 0.642 |
|  | Ammonium * Phosphate * Nitrate | 556.7 |  |  |
|  | Human land cover * Temperature | 553.5 | 0.593 | 0.365 |
|  | Phosphate * Human land cover * Season | 553.3 |  |  |
|  | Ammonium * Rainfall * Season | 556.1 | 0.824 | 0.642 |
|  | Human land cover * Ammonium * Temperature | 553.7 | 0.785 | 0.653 |
|  | Human land cover * Ammonium * Rainfall | 549.5 | 0.801 | 0.727 |
|  | Ammonium * Phosphate * Nitrate | 556.7 |  |  |
|  | Ammonium * Nitrate * Season | 573.5 | 0.983 | 0.134 |
|  | Phosphate * Temperature * Season | 562.5 |  |  |
|  | Phosphate * Human land cover * Season | 553.3 |  |  |
|  | Phosphate * Rainfall * Season | 559.6 |  |  |
|  | Temperature * Rainfall * Season | 557.5 |  |  |
|  | Temperature * Human land cover * Season | 563.6 | 0.629 | 0.408 |
|  | Human land cover * Season * Rainfall | 552.5 |  |  |
| EPT richness | Intercept | 211.2 | 0.503 | 0.000 |
|  | Human land cover | 208.6 | 0.524 | 0.193 |
|  | Phosphate | 213.0 | 0.528 | 0.007 |
|  | Nitrate | 212.1 | 0.512 | 0.023 |
|  | Ammonium | 200.2 | 0.540 | 0.382 |
|  | Temperature | 213.3 | 0.515 | 0.008 |
|  | Rainfall | 213.0 | 0.497 | 0.007 |
|  | Season | 213.4 | 0.503 | 0.001 |
|  | Ammonium * Season | 204.7 | 0.540 | 0.381 |
|  | Phosphate * Season | 217.9 | 0.517 | 0.010 |
|  | Nitrate * Season | 216.9 | 0.516 | 0.028 |
|  | Temperature * Season | 217.7 | 0.519 | 0.013 |
|  | Human land cover * Season | 213.4 | 0.526 | 0.198 |
|  | Rainfall * Season | 216.2 | 0.481 | 0.051 |
|  | Human land cover * Phosphate | 213.1 | 0.536 | 0.190 |
|  | Human land cover * Nitrate | 211.0 | 0.549 | 0.250 |
|  | Ammonium * Nitrate | 204.3 | 0.542 | 0.392 |
|  | Ammonium * Phosphate | 203.5 | 0.583 | 0.454 |
|  | Nitrate * Phosphate | 216.9 | 0.505 | 0.032 |
|  | Human land cover * Ammonium | 202.0 | 0.626 | 0.516 |
|  | Human land cover * Temperature | 213.4 | 0.525 | 0.212 |
|  | Human land cover * Rainfall | 212.3 | 0.539 | 0.250 |
|  | Phosphate * Rainfall | 216.7 | 0.483 | 0.029 |
|  | Nitrate * Temperature | 216.6 | 0.510 | 0.043 |
|  | Nitrate * Rainfall | 216.4 | 0.492 | 0.048 |
|  | Ammonium * Temperature | 203.8 | 0.543 | 0.410 |
|  | Ammonium * Rainfall | 203.5 | 0.567 | 0.429 |
|  | Human land cover * Phosphate * Temperature | 223.5 | 0.544 | 0.228 |
|  | Human land cover * Phosphate * Rainfall | 222.6 | 0.538 | 0.253 |
|  | Human land cover * Nitrate * Temperature | 220.0 | 0.551 | 0.371 |
|  | Human land cover * Nitrate * Rainfall | 219.5 | 0.571 | 0.346 |
|  | Human land cover * Ammonium * Temperature | 207.0 | 0.731 | 0.698 |
|  | Ammonium * Human land cover * Season | 206.4 | 0.730 | 0.639 |
|  | Ammonium * Rainfall * Season | 206.5 | 0.753 | 0.701 |
|  | Ammonium * Phosphate * Nitrate | 211.4 | 0.635 | 0.517 |
|  | Human land cover * Temperature | 213.4 | 0.525 | 0.212 |
|  | Phosphate * Human land cover * Season | 224.2 | 0.534 | 0.194 |
|  | Ammonium * Rainfall * Season | 206.5 | 0.753 | 0.701 |
|  | Human land cover * Ammonium * Temperature | 207.0 | 0.731 | 0.698 |
|  | Human land cover * Ammonium * Rainfall | 206.7 | 0.751 | 0.673 |
|  | Ammonium * Phosphate * Nitrate | 211.4 | 0.635 | 0.517 |
|  | Ammonium * Nitrate * Season | 214.3 | 0.557 | 0.424 |
|  | Phosphate * Temperature * Season | 227.8 | 0.531 | 0.024 |
|  | Phosphate * Human land cover * Season | 224.2 | 0.534 | 0.194 |
|  | Phosphate * Rainfall * Season | 224.9 | 0.453 | 0.092 |
|  | Temperature * Rainfall * Season | 225.2 | 0.517 | 0.113 |
|  | Temperature * Human land cover * Season | 223.2 | 0.532 | 0.234 |
|  | Human land cover * Season * Rainfall | 221.1 | 0.574 | 0.357 |
| SDI | Intercept | 16.8 | 0.332 | 0.000 |
|  | Human land cover | 7.3 | 0.423 | 0.252 |
|  | Phosphate | 20.1 | 0.367 | 0.015 |
|  | Nitrate | 21.0 | 0.354 | 0.009 |
|  | Ammonium | 16.2 | 0.165 | 0.074 |
|  | Temperature | 17.0 | 0.445 | 0.062 |
|  | Rainfall | 17.5 | 0.320 | 0.024 |
|  | Season | 18.6 | 0.349 | 0.007 |
|  | Ammonium * Season | 17.1 | 0.202 | 0.164 |
|  | Phosphate * Season | 21.7 | 0.383 | 0.032 |
|  | Nitrate * Season | 26.0 | 0.360 | 0.010 |
|  | Temperature * Season | 15.3 | 0.534 | 0.138 |
|  | Human land cover * Season | 12.0 | 0.432 | 0.256 |
|  | Rainfall * Season | 21.9 | 0.256 | 0.043 |
|  | Human land cover * Phosphate | 11.3 | 0.420 | 0.269 |
|  | Human land cover * Nitrate | 11.3 | 0.420 | 0.269 |
|  | Ammonium * Nitrate | 15.8 | 0.220 | 0.182 |
|  | Ammonium * Phosphate | 15.7 |  |  |
|  | Nitrate * Phosphate | 24.3 | 0.389 | 0.047 |
|  | Human land cover * Ammonium | 11.0 | 0.316 | 0.276 |
|  | Human land cover * Temperature | 10.0 | 0.416 | 0.288 |
|  | Human land cover * Rainfall | 11.8 | 0.408 | 0.258 |
|  | Phosphate * Rainfall | 19.1 | 0.379 | 0.072 |
|  | Nitrate * Temperature | 22.2 | 0.555 | 0.102 |
|  | Nitrate * Rainfall | 24.2 | 0.305 | 0.041 |
|  | Ammonium * Temperature | 14.5 | 0.293 | 0.202 |
|  | Ammonium * Rainfall | 11.2 |  |  |
|  | Human land cover * Phosphate * Temperature | 20.4 | 0.454 | 0.072 |
|  | Human land cover * Phosphate * Rainfall | 14.1 | 0.471 | 0.361 |
|  | Human land cover * Nitrate * Temperature | 9.8 | 0.460 | 0.448 |
|  | Human land cover * Nitrate * Rainfall | 12.9 | 0.603 | 0.384 |
|  | Human land cover * Ammonium * Temperature | 12.9 |  |  |
|  | Ammonium * Human land cover * Season | 13.3 |  |  |
|  | Ammonium * Rainfall * Season | 10.4 |  |  |
|  | Ammonium * Phosphate * Nitrate | 23.0 | 0.581 | 0.267 |
|  | Human land cover * Temperature | 10.0 | 0.416 | 0.288 |
|  | Phosphate * Human land cover * Season | 9.55 | 0.511 | 0.361 |
|  | Ammonium * Rainfall * Season | 10.4 |  |  |
|  | Human land cover * Ammonium * Temperature | 13.3 |  |  |
|  | Human land cover * Ammonium * Rainfall |  |  |  |
|  | Ammonium * Phosphate * Nitrate | 23.0 | 0.581 | 0.267 |
|  | Ammonium * Nitrate * Season | 26.0 | 0.246 | 0.195 |
|  | Phosphate * Temperature * Season | 23.2 | 0.554 | 0.159 |
|  | Phosphate * Human land cover * Season | 19.3 | 0.511 | 0.361 |
|  | Phosphate * Rainfall * Season | 28.6 | 0.255 | 0.132 |
|  | Temperature * Rainfall * Season | 21.8 | 0.353 | 0.247 |
|  | Temperature * Human land cover * Season | 15.0 | 0.601 | 0.350 |
|  | Human land cover * Season * Rainfall | 16.1 | 0.503 | 0.347 |
| Mean body size | Intercept | 104.4 | 0.359 | 0.000 |
|  | Human land cover | 106.1 | 0.323 | 0.034 |
|  | Phosphate | 106.7 | 0.360 | 0.000 |
|  | Nitrate | 106.1 | 0.371 | 0.012 |
|  | Ammonium | 106.5 | 0.361 | 0.007 |
|  | Temperature | 105.7 | 0.333 | 0.038 |
|  | Rainfall | 99.8 | 0.402 | 0.141 |
|  | Season | 100.7 | 0.413 | 0.113 |
|  | Ammonium * Season | 104.9 | 0.419 | 0.132 |
|  | Phosphate * Season | 105.6 | 0.420 | 0.112 |
|  | Nitrate * Season | 102.3 | 0.463 | 0.174 |
|  | Temperature * Season | 104.1 | 0.387 | 0.159 |
|  | Human land cover * Season | 104.8 | 0.368 | 0.173 |
|  | Rainfall * Season | 102.5 | 0.463 | 0.167 |
|  | Human land cover * Phosphate | 108.8 | 0.397 | 0.084 |
|  | Human land cover * Nitrate | 109.6 | 0.374 | 0.051 |
|  | Ammonium * Nitrate | 110.8 | 0.374 | 0.019 |
|  | Ammonium * Phosphate | 108.3 | 0.440 | 0.070 |
|  | Nitrate * Phosphate | 110.9 | 0.387 | 0.016 |
|  | Human land cover * Ammonium | 109.2 | 0.316 | 0.095 |
|  | Human land cover * Temperature | 108.6 | 0.349 | 0.110 |
|  | Human land cover * Rainfall | 95.9 |  |  |
|  | Phosphate * Rainfall | 104.4 | 0.391 | 0.149 |
|  | Nitrate * Temperature | 109.4 | 0.352 | 0.061 |
|  | Nitrate * Rainfall | 104.1 | 0.414 | 0.154 |
|  | Ammonium * Temperature | 110.6 | 0.337 | 0.039 |
|  | Ammonium * Rainfall | 104.5 | 0.400 | 0.145 |
|  | Human land cover * Phosphate * Temperature | 115.1 | 0.454 | 0.202 |
|  | Human land cover * Phosphate * Rainfall | 105.0 |  |  |
|  | Human land cover * Nitrate * Temperature | 117.5 | 0.405 | 0.147 |
|  | Human land cover * Nitrate * Rainfall | 106.4 |  |  |
|  | Human land cover * Ammonium * Temperature | 116.1 | 0.325 | 0.237 |
|  | Ammonium * Human land cover * Season | 115.9 | 0.325 | 0.237 |
|  | Ammonium * Rainfall * Season | 97.5 |  |  |
|  | Ammonium * Phosphate * Nitrate | 118.9 | 0.439 | 0.084 |
|  | Human land cover * Temperature | 108.6 | 0.349 | 0.110 |
|  | Phosphate * Human land cover * Season | 114.3 | 0.426 | 0.191 |
|  | Ammonium * Rainfall * Season | 108.4 | 0.512 | 0.287 |
|  | Human land cover * Ammonium * Temperature | 116.1 | 0.325 | 0.237 |
|  | Human land cover * Ammonium * Rainfall | 102.3 |  |  |
|  | Ammonium * Phosphate * Nitrate | 118.9 | 0.439 | 0.084 |
|  | Ammonium * Nitrate * Season | 111.1 | 0.537 | 0.230 |
|  | Phosphate * Temperature * Season | 112.8 | 0.409 | 0.219 |
|  | Phosphate * Human land cover * Season | 114.3 | 0.426 | 0.191 |
|  | Phosphate * Rainfall * Season | 109.0 | 0.532 | 0.231 |
|  | Temperature * Rainfall * Season | 114.7 | 0.423 | 0.234 |
|  | Temperature * Human land cover * Season | 103.3 |  |  |
|  | Human land cover * Season * Rainfall | 101.9 |  |  |
| Community biomass | Intercept | 619.6 | 0.588 | 0.000 |
|  | Human land cover | 621.7 | 0.543 | 0.028 |
|  | Phosphate | 621.3 | 0.553 | 0.021 |
|  | Nitrate | 621.4 | 0.611 | 0.013 |
|  | Ammonium | 621.9 | 0.575 | 0.007 |
|  | Temperature | 617.4 | 0.490 | 0.122 |
|  | Rainfall | 619.9 | 0.584 | 0.041 |
|  | Season | 619.4 | 0.579 | 0.048 |
|  | Ammonium * Season | 624.3 | 0.569 | 0.060 |
|  | Phosphate * Season | 623.3 | 0.510 | 0.097 |
|  | Nitrate * Season | 622.3 | 0.598 | 0.087 |
|  | Temperature * Season | 620.3 | 0.560 | 0.172 |
|  | Human land cover * Season | 623.8 | 0.516 | 0.093 |
|  | Rainfall * Season | 620.3 | 0.599 | 0.147 |
|  | Human land cover * Phosphate | 623.4 | 0.576 | 0.122 |
|  | Human land cover * Nitrate | 626.3 | 0.541 | 0.045 |
|  | Ammonium * Nitrate | 625.3 | 0.553 | 0.073 |
|  | Ammonium * Phosphate | 622.4 | 0.566 | 0.081 |
|  | Nitrate * Phosphate | 625.0 | 0.545 | 0.062 |
|  | Human land cover * Ammonium | 620.2 | 0.592 | 0.211 |
|  | Human land cover * Temperature | 619.3 | 0.638 | 0.242 |
|  | Human land cover * Rainfall | 623.1.5 | 0.317 | 0.269 |
|  | Phosphate * Rainfall | 624.1 | 0.508 | 0.090 |
|  | Nitrate * Temperature | 624.1 | 0.483 | 0.146 |
|  | Nitrate * Rainfall | 624.6 | 0.603 | 0.048 |
|  | Ammonium * Temperature | 624.9 | 0.485 | 0.127 |
|  | Ammonium * Rainfall | 623.7 | 0.522 | 0.522 |
|  | Human land cover * Phosphate * Temperature | 621.6 | 0.666 | 0.403 |
|  | Human land cover * Phosphate * Rainfall | 630.1 | 0.529 | 0.241 |
|  | Human land cover * Nitrate * Temperature | 626.4 | 0.831 | 0.251 |
|  | Human land cover * Nitrate * Rainfall | 625.4 |  |  |
|  | Human land cover * Ammonium * Temperature | 618.7 |  |  |
|  | Ammonium * Human land cover * Season | 622.4 | 0.543 | 0.450 |
|  | Ammonium * Rainfall * Season | 629.7 | 0.611 | 0.213 |
|  | Ammonium * Phosphate * Nitrate | 633.7 | 0.566 | 0.101 |
|  | Human land cover * Temperature | 619.3 | 0.638 | 0.242 |
|  | Phosphate * Human land cover * Season | 631.2 | 0.545 | 0.171 |
|  | Ammonium * Rainfall * Season | 629.7 | 0.611 | 0.213 |
|  | Human land cover * Ammonium * Temperature | 618.7 |  |  |
|  | Human land cover * Ammonium * Rainfall | 624.7 | 0.597 | 0.455 |
|  | Ammonium * Phosphate * Nitrate | 633.7 | 0.566 | 0.101 |
|  | Ammonium * Nitrate * Season | 630.4 | 0.632 | 0.206 |
|  | Phosphate * Temperature * Season | 631.4 | 0.563 | 0.186 |
|  | Phosphate * Human land cover * Season | 623.3 | 0.510 | 0.097 |
|  | Phosphate * Rainfall * Season | 630.1 | 0.609 | 0.180 |
|  | Temperature * Rainfall * Season | 624.8 | 0.600 | 0.258 |
|  | Temperature * Human land cover * Season | 625.4 | 0.752 | 0.347 |
|  | Human land cover * Season * Rainfall | 626.4 | 0.449 | 0.329 |

**Table S3:** Best-fit models, distribution and random effect choices for each of the seven response variables.

| Response variable | Best-fit model | Distribution | Retained random effect(s) |
| --- | --- | --- | --- |
| Taxa abundance | Human land cover * ammonium | Negative binomial | Site and ecoregion |
| Taxa richness | Intercept | Poisson | Site and ecoregion |
| EPT abundance | Human land cover * ammonium | Negative binomial | Site and ecoregion |
| EPT richness | Ammonium | Poisson | Ecoregion |
| Simpson’s diversity index | Human land cover | Gamma with a log-link | Site |
| Biomass | Temperature | Gamma with a log-link | Site |
| Mean body size | Human land cover * rainfall | Gamma with a log-link | NA |

**Table S4:** Site information for the 30 sites

| **Site name and season** | **Water Temperature** | **Season** | **pH** | **Ammonium** | **Conductivity** | **Width** | **Depth** | **Phosphate** | **Nitrate** | **TDS** |
| --- | --- | --- | --- | --- | --- | --- | --- | --- | --- | --- |
| Apies | 20.43 | Hot-wet | 7.89 | 4.43 | 480.00 | 6.00 | 40.00 | 3.97 | 61.55 | 136.00 |
| Apies | 18.60 | Cold-dry | 7.81 | 9.76 | 455.00 | 9.00 | 40.00 | 61.80 | 4.73 | 377.33 |
| Bloemspruit | 15.80 | Hot-wet | 7.71 | - | 730.33 | 3 | 39 | - | - | 339.0 |
| Bloemspruit | 16.33 | Cold-dry | 7.55 | 22.1 | 868.67 | 2.50 | 40.00 | 11.30 | 9.47 | 434.67 |
| Bloukrans | 17.77 | Hot-wet | 7.61 | 32.14 | 1557.67 | 1.50 | 0.00 | 18.27 | 30.80 | 679.33 |
| Bloukrans | 14.87 | Cold-dry | 7.72 | 31.82 | 716.67 | 1.50 | 20.00 | 15.83 | 5.10 | 651.33 |
| Braamspruit | 18.37 | Hot-wet | 7.71 | 0 | 332.00 | 5.00 | 30.00 | 34.30 | 93.80 | 88.67 |
| Braamspruit | 14.20 | Cold-dry | 8.36 | 8.17 | 199.00 | 6.00 | 35.00 | 8.70 | 1.53 | 0.00 |
| Buffalo | 20.59 | Hot-wet | 7.83 | 0.13 | 550.33 | 8 | 20 | 8.5 | 40.17 | 290 |
| Buffalo | 18.30 | Cold-dry | 8.00 | 6.84 | 849.33 | 6.00 | 35.00 | 7.40 | 19.77 | 429.67 |
| Hennops | 19.30 | Hot-wet | 7.74 | 11 | 771.67 | 6.00 | 20.00 | 6.77 | 81.50 | 231.67 |
| Hennops | 16.17 | Cold-dry | 7.83 | 40.13 | 753.67 | 10.00 | 80.00 | 13.20 | 35.83 | 376.33 |
| Igoda | 18.27 | Hot-wet | 7.81 | 6.14 | 2087.67 | 3.00 | 17.00 | 13.67 | 0.00 | 775.00 |
| Igoda | 15.80 | Cold-dry Cold-dry | 7.92 | 35.03 | 2058.00 | 1.00 | 15.00 | 59.23 | 0.17 | 1027.33 |
| Kat | 20.00 | Hot-wet | 7.58 | 3.07 | 352.33 | 2.00 | 31.00 | 56.30 | 31.70 | 191.67 |
| Kat | 14.50 | Cold-dry | 7.92 | 1.41 | 612.67 | 2.00 | 26.00 | 40.80 | 1.00 | 305.33 |
| Keiskamma | 17.87 | Hot-wet | 7.85 | 1.45 | 157.00 | 6.00 | 42.00 | 98.50 | 87.93 | 97.67 |
| Keiiskamma | 16.03 | Cold-dry | 8.16 | 0 | 262.00 | 4.00 | 28.00 | 4.50 | 5.57 | 132.33 |
| Klipriviersberg | 18.57 | Hot-wet | 7.82 | 33.92 | 308.00 | 2.00 | 28.00 | 3.50 | 54.25 | 137.00 |
| Klipriviersberg | 15.70 | Cold-dry | 8.44 | 1.3 | 380.00 | 2.00 | 25.00 | 15.47 | 2.97 | 140.00 |
| Letaba | 31.60 | Hot-wet | 7.02 | 0.2 | 464.10 | 25.00 | 30.00 | 3.13 | 1.60 | 305.15 |
| Letaba | 18.00 | Cold-dry | 7.03 | 1.26 | 407.67 | 24.00 | 40.90 | 0.00 | 19.07 | 148.33 |
| Lisbon | 18.70 | Hot-wet Hot-wet | 6.82 | 2.53 | 23.31 | 3.00 | 45.00 | 4.07 | 9.09 | 8.77 |
| Lisbon | 10.80 | Cold-dry | 8.16 | 1.1 | 29.86 | 3.00 | 45.00 | 1.10 | 7.43 | 7.25 |
| Luvuvu | 19.37 | Hot-wet | 6.92 | 1.86 | 142.00 | 40.00 | 50.00 | 1.80 | 2.33 | 69.86 |
| Luvuvu | 20.20 | Cold-dry | 7.04 | 1.49 | 219.27 | 30.00 | 40.00 | 1.40 | 1.60 | 87.55 |
| Mac-mac | 17.85 | Hot-wet | 6.91 | 0.56 | 165.30 | 0.00 | 20.00 | 10.93 | 7.57 | 60.57 |
| Mac-mac | 11.20 | Cold-dry | 8.22 | 1 | 176.50 | 4.00 | 20.00 | 79.70 | 11.20 | 44.38 |
| Modderriver | 15.93 | Hot-wet | 7.97 | - | 687.33 | 2 | 28 |  |  | 158.33 |
| Modderriver | 14.37 | Cold-dry | 8.49 | 7.36 | 484.33 | 3.00 | 40.00 | 4.13 | 12.13 | 243.33 |
| Muldersdrift | 17.97 | Hot-wet | 7.77 | 2.76 | 188.67 | 5.00 | 60.00 | 7.10 | 21.20 | 69.33 |
| Muldersdrift | 13.00 | Cold-dry | 8.09 | 2.69 | 261.33 | 8.00 | 60.00 | 4.00 | 6.33 | 134.33 |
| Mvudi | 19.75 | Hot-wet | 7.00 | 0 | 219.50 | 0.00 | 30.00 | 0.00 | 0.00 | 110.00 |
| Mvudi | - | Cold-dry |  | - | - | - | - | - | - | - |
| Ncera | 17.90 | Hot-wet | 7.97 | 13.53 | 1131.67 | 4.00 | 40.00 | 11.73 | 62.30 | 497.00 |
| Ncera | 15.63 | Cold-dry | 8.30 | 6.9 | 995.33 | 3.00 | 30.00 | 19.77 | 1.17 | 498.00 |
| Olifants | 21.27 | Hot-wet | 7.52 | 22.91 | 863.67 | 4.00 | 30.00 | 16.30 | 69.25 | 233.00 |
| Olifants | 17.97 | Cold-dry | 7.58 | 55.56 | 863.67 | 3.00 | 50.00 | 16.17 | 3.43 | 0.00 |
| Regu | 19.97 | Hot-wet | 7.56 | 3.14 | 1897.33 | 2.00 | 13.00 | 69.10 | 26.60 | 716.33 |
| Regu | 19.57 | Cold-dry | 7.52 | 1.18 | 1980.00 | 1.50 | 20.00 | 58.80 | 9.57 | 980.00 |
| Renospruit | 16.47 | Hot-wet | 7.60 | 0 | 713.00 | 3.00 | 38.00 | 0.00 | 0.00 | 299.33 |
| Renospruit | 13.67 | Cold-dry | 7.79 | 38.56 | 754.67 | 10.00 | 70.00 | 9.00 | 14.57 | 377.33 |
| Sands | 18.23 | Hot-wet | 7.85 | 0 | 743.00 | 0.00 | 60.00 | 0.00 | 0.00 | 201.33 |
| Sands | 13.41 | Cold-dry | 7.93 | 8.33 | 1378.67 | 11 | 60 | 15.57 | 16.40 | 691.67 |
| Selati | 25.10 | Hot-wet | 6.99 | 6.96 | 2237.67 | 8.00 | 20.00 | 10.90 | 7.97 | 1063.67 |
| Selati | 18.93 | Cold-dry | 7.02 | 0.96 | 2414.33 | 2.00 | 30.00 | 2.77 | 23.90 | 915.27 |
| Shingwedzi | 28.75 | Hot-wet | 6.88 | 4.74 | 1684.67 | 15.00 | 30.00 | 23.63 | 16.70 | 1.29 |
| Shingwedzi | 22.66 | Cold-dry | 7.03 | 4.64 | 1684.67 | 0.00 | 30.00 | 0.00 | 18.17 | 596.69 |
| Skinnerspruit | 21.27 | Hot-wet | 7.47 | 22.16 | 745.00 | 2.00 | 30.00 | 10.73 | 84.30 | 234.67 |
| Skinnerspruit | 16.67 | Cold-dry | 7.74 | 7.41 | 556.00 | 4.00 | 60.00 | 3.33 | 3.10 | 279.67 |
| Treur | 20.23 | Hot-wet | 7.07 | 0.2 | 46.68 | 8.00 | 60.00 | 0.00 | 6.83 | 19.16 |
| Treur | 16.60 | Cold-dry | 7.02 | 0.2 | 78.41 | 0.00 | 0.00 | 3.20 | 10.13 | 21.99 |
| Tyume | 16.70 | Hot-wet | 7.97 | 3.93 | 77.00 | 5.00 | 20.00 | 14.47 | 30.30 | 53.67 |
| Tyume | 15.30 | Cold-dry | 8.35 | 0 | 75.33 | 5.00 | 30.00 | 4.77 | 0.60 | 38.33 |
| Vietvier | 17.07 | Hot-wet |  | - | - | - | - | - | - | - |
| Vietvier | 13.97 | Cold-dry | 8.13 | 0.47 | 476.00 | 6.00 | 70.00 | 13.27 | 19.37 | 240.00 |
| Walkerspruit | 18.60 | Hot-wet | 7.75 | 0 | 304.00 | 2.00 | 30.00 | 2.33 | 0.00 | 129.67 |
| Walkerspruit | 16.27 | Cold-dry | 8.08 | 3.25 | 346.00 | 2.00 | 30.00 | 2.35 | 4.10 | 167.00 |
| Mambedi | 14.33 | Cold-dry | 7.46 | 0.3 | 528.73 | 2.50 | 0.40 | 2.70 | 0.05 | 152.30 |

**Table S5**: Randomisation test of the main effects

| Predictor | *df* | R^2^ | F-value | p-value |
| --- | --- | --- | --- | --- |
| Human land cover | 1 | 0.052 | 2.872 | **0.002**** |
| Ammonium | 1 | 0.032 | 1.720 | 0.063 |
| Temperature | 1 | 0.016 | 0.838 | 0.595 |
| Rainfall | 1 | 0.010 | 0.516 | 0.914 |
| Phosphate | 1 | 0.015 | 0.808 | 0.628 |
| Nitrate | 1 | 0.017 | 0.888 | 0.543 |
| Season | 1 | 0.011 | 0.559 | 0.885 |
| Conductivity | 1 | 0.023 | 1.218 | 0.257 |
| TDS | 1 | 0.025 | 1.308 | 0.200 |
| Width | 1 | 0.037 | 2.024 | **0.0236 *** |
| Depth | 1 | 0.034 | 1.805 | 0.0527 |
| pH | 1 | 0.057 | 3.117 | **<0.001 ***** |
| Water temperature | 1 | 0.021 | 1.140 | 0.319 |

**Figure S2:** Ammonium effects on mean biomass.

**Figure S3:** ammonium, rainfall and season interaction on mean body size.

**Table S6: Taxa contributing to biomass increase with ammonium**

| Family | r_biomass |  |
| --- | --- | --- |
| Hydraenidae | -1 | Very strong |
| Libellulidae | -1 | Very strong |
| Aphelocheiridae | -1 | Very strong |
| Corydalidae | -1 | Very strong |
| Dytiscidae | -1 | Very strong |
| Hydroptilidae | -1 | Very strong |
| Micronectidae | 1 | Very strong |
| Athericidae | 0.5 | Strong |
| Blephariceridae | 0.5 | Strong |
| Corixidae | 0.5 | Strong |
| Perlidae | -0.5 | Strong |
| Polymitarcyidae | -0.5 | Strong |
| Chironomidae | 0.4375557 | Weak |
| Lestidae | -0.4 | Weak |
| Simulidae | -0.3130165 | Moderate |
| Heptageniidae | -0.2874303 | Weak |
| Caenidae | 0.20180406 | Weak |
| Elmidae | 0.19365765 | Weak |
| Leptophlebiidae | -0.1751565 | Weak |
| Aeshnidae | 0.1 | Weak |
| Tabanidae | -0.1 | Weak |
| Hydropsychidae | 0.06726017 | Weak |

**Table S7:** Taxa contribution to the interactive effects of ammonium and human land cover

| Taxa | average | sd | ratio | ava | avb | cumsum | p |
| --- | --- | --- | --- | --- | --- | --- | --- |
| Chironomidae | 1.92E-01 | 0.190859 | 1.006889 | 54.33333 | 61.54545 | 0.244859 | 0.381 |
| Simulidae | 1.52E-01 | 0.20721 | 0.73397 | 22.41667 | 121.1818 | 0.438639 | 0.297 |
| Caenidae | 1.36E-01 | 0.158303 | 0.859914 | 29.91667 | 34.18182 | 0.612084 | 0.074 |
| Baetidae | 1.30E-01 | 0.127426 | 1.016365 | 36.5 | 28.72727 | 0.777102 | 0.94 |
| Oligochaeta | 5.31E-02 | 0.118983 | 0.446193 | 12.08333 | 9.545455 | 0.844746 | 0.133 |
| Hydropsychidae | 3.61E-02 | 0.068802 | 0.525071 | 16.5 | 4.090909 | 0.890775 | 0.732 |
| Elmidae | 2.95E-02 | 0.053209 | 0.555317 | 13.75 | 1.727273 | 0.928424 | 0.368 |
| Leptophlebiidae | 1.01E-02 | 0.01556 | 0.647601 | 3.333333 | 1.090909 | 0.941263 | 0.977 |
| Heptageniidae | 5.77E-03 | 0.029452 | 0.195908 | 0 | 0.363636 | 0.948615 | 0.817 |
| Limoniidae | 5.77E-03 | 0.016681 | 0.345794 | 0 | 0.818182 | 0.955965 | 0.066 |
| Notonemouridae | 5.51E-03 | 0.021801 | 0.252667 | 0 | 0.636364 | 0.962983 | 0.231 |
| Hirudinea | 5.49E-03 | 0.014119 | 0.388811 | 3.666667 | 1.545455 | 0.969978 | 0.135 |
| Hydroptilidae | 4.12E-03 | 0.009232 | 0.446749 | 0.916667 | 1.363636 | 0.975233 | 0.09 |
| Tipulidae | 1.93E-03 | 0.007273 | 0.266031 | 0 | 0.363636 | 0.977698 | 0.169 |
| Nematoda | 1.75E-03 | 0.006471 | 0.271178 | 0.583333 | 0 | 0.979934 | 0.381 |
| Tabanidae | 1.71E-03 | 0.00403 | 0.425176 | 0.583333 | 0.181818 | 0.982117 | 0.553 |
| Lestidae | 1.64E-03 | 0.005439 | 0.300679 | 0 | 0.363636 | 0.984201 | 0.546 |
| Naucoridae | 1.59E-03 | 0.008572 | 0.185406 | 0 | 0.090909 | 0.986226 | 0.074 |
| Polymitarcyidae | 1.32E-03 | 0.003967 | 0.333044 | 0 | 0.181818 | 0.987909 | 0.244 |
| Glossiphoniidae | 1.00E-03 | 0.003698 | 0.271178 | 0.333333 | 0 | 0.989187 | 0.68 |
| Libellulidae | 8.37E-04 | 0.00364 | 0.230096 | 0 | 0.090909 | 0.990254 | 0.957 |
| Perlidae | 8.37E-04 | 0.00364 | 0.230096 | 0 | 0.090909 | 0.991321 | 0.538 |
| Hydraenidae | 8.34E-04 | 0.001828 | 0.45638 | 0.5 | 0 | 0.992384 | 0.477 |
| Micronectidae | 7.57E-04 | 0.00272 | 0.278396 | 0 | 0.181818 | 0.993349 | 0.285 |
| Ephemerellidae | 7.29E-04 | 0.001939 | 0.375775 | 0 | 0.181818 | 0.994277 | 0.465 |
| Aeshnidae | 7.10E-04 | 0.001625 | 0.436709 | 0.083333 | 0.181818 | 0.995181 | 0.522 |
| Ancylinae | 6.84E-04 | 0.002635 | 0.259724 | 0.166667 | 0 | 0.996053 | 0.371 |
| Corixidae | 6.40E-04 | 0.001451 | 0.441058 | 0.166667 | 0.181818 | 0.996869 | 0.284 |
| Hydrophilidae | 4.01E-04 | 0.001401 | 0.286256 | 0.25 | 0 | 0.99738 | 0.775 |
| Planorbidae | 3.86E-04 | 0.001327 | 0.291073 | 0.333333 | 0 | 0.997872 | 0.663 |
| Culicidae | 3.79E-04 | 0.00136 | 0.278396 | 0 | 0.090909 | 0.998354 | 0.532 |
| Philopotamidae | 3.79E-04 | 0.00136 | 0.278396 | 0 | 0.090909 | 0.998837 | 0.517 |
| Dytiscidae | 3.42E-04 | 0.001318 | 0.259724 | 0.083333 | 0 | 0.999273 | 0.614 |
| Corydalidae | 1.84E-04 | 0.000433 | 0.424812 | 0.083333 | 0.090909 | 0.999507 | 0.505 |
| Velidae | 1.57E-04 | 0.000553 | 0.283263 | 0.083333 | 0 | 0.999707 | 0.407 |
| Blephariceridae | 1.34E-04 | 0.000467 | 0.286256 | 0.083333 | 0 | 0.999877 | 0.909 |
| Scirtidae | 9.66E-05 | 0.000332 | 0.291073 | 0.083333 | 0 | 1 | 0.406 |
| Ampullariidae | 0.00E+00 | 0 | NaN | 0 | 0 | 1 | *NA* |
| Aphelocheiridae | 0.00E+00 | 0 | NaN | 0 | 0 | 1 | *NA* |
| Athericidae | 0.00E+00 | 0 | NaN | 0 | 0 | 1 | *NA* |
| Beetle.X | 0.00E+00 | 0 | NaN | 0 | 0 | 1 | *NA* |
| Crambidae | 0.00E+00 | 0 | NaN | 0 | 0 | 1 | *NA* |
| Cyrenidae | 0.00E+00 | 0 | NaN | 0 | 0 | 1 | *NA* |
| Ephemeridae | 0.00E+00 | 0 | NaN | 0 | 0 | 1 | *NA* |
| Ephemerythidae | 0.00E+00 | 0 | NaN | 0 | 0 | 1 | *NA* |
| Gammaridae | 0.00E+00 | 0 | NaN | 0 | 0 | 1 | *NA* |
| Lampyridae | 0.00E+00 | 0 | NaN | 0 | 0 | 1 | *NA* |
| Leptoceridae | 0.00E+00 | 0 | NaN | 0 | 0 | 1 | *NA* |
| Muscidae | 0.00E+00 | 0 | NaN | 0 | 0 | 1 | *NA* |
| Naididae | 0.00E+00 | 0 | NaN | 0 | 0 | 1 | *NA* |
| Nepidae | 0.00E+00 | 0 | NaN | 0 | 0 | 1 | *NA* |
| Notonectidae | 0.00E+00 | 0 | NaN | 0 | 0 | 1 | *NA* |
| Oligoneuriidae | 0.00E+00 | 0 | NaN | 0 | 0 | 1 | *NA* |
| Pomatiopsidae | 0.00E+00 | 0 | NaN | 0 | 0 | 1 | *NA* |
| Thiaridae | 0.00E+00 | 0 | NaN | 0 | 0 | 1 |  |
